# Attractive targeted sugar bait phase III trials in Kenya, Mali, and Zambia

**DOI:** 10.1186/s13063-022-06555-8

**Published:** 2022-08-09

**Authors:** Attractive Targeted Sugar Bait Phase III Trial Group, Thomas P. Eisele, Immo Kleinschmidt, Sophie Sarrassat, Feiko terKuile, John Miller, Javan Chanda, Kafula Silumbe, Aaron Samuels, Julia Janssen, Caroline Ogwang, John Bradley, Erica Orange, Josh Yukich, Ruth Ashton, Irene Kyomuhangi, Angela F. Harris, Seydou Doumbia, Mahamoudou Toure, Mohamed Moumine, Silas Majambere, Monicah Mirai Mburu, Gift Mwaanga, Limonty Simubali, Edgar Simulundu, Adam Bennett, Laurence Slutsker, Gunter Muller, Eric Ochomo, John Gimnig, Paul C. D. Johnson, Joseph Wagman, Megan Littrell

**Affiliations:** 1 Center for Applied Malaria Research and Evaluation, Department of Tropical Medicine, Tulane University School of Public Health and Tropical Medicine, New Orleans, LA 70125 USA; 2 MRC International Statistics and Epidemiology Group, London School of Hygiene and Tropical Medicine, London, UK; 3 Wits Research Institute for Malaria, School of Pathology, Faculty of Health Sciences, University of the Witwatersrand, Johannesburg, South Africa; 4 Southern African Development Community Malaria Elimination Eight Secretariat, Windhoek, Namibia; 5 Centre for Maternal, Adolescent, Reproductive and Child Health (MARCH), London School of Hygiene and Tropical Medicine, London, UK; 6 Department of Clinical Sciences, Liverpool School of Tropical Medicine, Liverpool, UK; 7 Centre for Global Health Research, Kenya Medical Research Institute, Kisumu, Kenya; 8 PATH, Lusaka, Zambia; 9 Malaria Branch, Division of Parasitic Diseases and Malaria, Center for Global Health, Centers for Disease Control and Prevention, Kisumu, Kenya; 10 Malaria Branch, Division of Parasitic Diseases and Malaria, Center for Global Health, Centers for Disease Control and Prevention, Atlanta, GA USA; 11 Epidemic Intelligence Service, U.S. Centers for Disease Control and Prevention, Atlanta, GA USA; 12 PATH, Seattle, WA USA; 13 IVCC, Liverpool School of Tropical Medicine, Liverpool, UK; 14 University Clinical Research Center (UCRC), and Malaria Research and Training Center (MRTC), Bamako, Mali; 15 University of Sciences, Techniques and Technology of Bamako (USTTB), Bamako, Mali; 16 Macha Research Trust, Choma, Zambia; 17 Independent Consultant, Atlanta, GA USA; 18 UniversityInstitute of Biodiversity Animal Health and Comparative Medicine, University of Glasgow, Glasgow, UK; 19 PATH, Washington DC, USA

**Keywords:** Malaria, Vector control, Attractive targeted sugar bait, Cluster-randomized controlled trial

## Abstract

**Background:**

Long-lasting insecticidal nets (LLINs) and indoor residual spraying (IRS) target night-time indoor biting mosquitoes and effectively reduce malaria transmission in rural settings across Africa, but additional vector control tools are needed to interrupt transmission. Attractive targeted sugar baits (ATSBs) attract and kill mosquitoes, including those biting outdoors. Deployment of ATSBs incorporating the insecticide dinotefuran was associated with major reductions in mosquito density and longevity in Mali. The impact of this promising intervention on malaria transmission and morbidity now needs to be determined in a range of transmission settings.

**Methods/design:**

We will conduct three similar stand-alone, open-label, two-arm, cluster-randomized, controlled trials (cRCTs) in Mali, Kenya, and Zambia to determine the impact of ATSB + universal vector control versus universal vector control alone on clinical malaria. The trials will use a “fried-egg” design, with primary outcomes measured in the core area of each cluster to reduce spill-over effects. All household structures in the ATSB clusters will receive two ATSBs, but the impact will be measured in the core of clusters. Restricted randomization will be used. The primary outcome is clinical malaria incidence among children aged 5–14 years in Mali and 1–14 years in Kenya and Zambia. A key secondary outcome is malaria parasite prevalence across all ages. The trials will include 76 clusters (38 per arm) in Mali and 70 (35 per arm) in each of Kenya and Zambia. The trials are powered to detect a 30% reduction in clinical malaria, requiring a total of 3850 person-years of follow-up in Mali, 1260 person-years in Kenya, and 1610 person-years in Zambia. These sample sizes will be ascertained using two seasonal 8-month cohorts in Mali and two 6-month seasonal cohorts in Zambia. In Kenya, which has year-round transmission, four 6-month cohorts will be used (total 24 months of follow-up). The design allows for one interim analysis in Mali and Zambia and two in Kenya.

**Discussion:**

Strengths of the design include the use of multiple study sites with different transmission patterns and a range of vectors to improve external validity, a large number of clusters within each trial site, restricted randomization, between-cluster separation to minimize contamination between study arms, and an adaptive trial design. Noted threats to internal validity include open-label design, risk of contamination between study arms, risk of imbalance of covariates across study arms, variation in durability of ATSB stations, and potential disruption resulting from the COVID-19 pandemic.

**Trial registration:**

Zambia: ClinicalTrials.gov NCT04800055. Registered on March 15, 2021

Mali: ClinicalTrials.gov NCT04149119. Registered on November 4, 2019

Kenya: ClinicalTrials.gov NCT05219565. Registered on February 2, 2022

**Supplementary Information:**

The online version contains supplementary material available at 10.1186/s13063-022-06555-8.

## Background

Indoor residual spraying of insecticides [IRS] and long-lasting insecticidal nets [LLINs] target indoor, night-time-biting, and indoor-resting mosquitoes and have contributed to a 68% reduction in malaria parasite prevalence in Africa between 2000 and 2015 [1]. However, their effectiveness is threatened by the development of insecticide resistance [2] and changes in vector bionomics, including relative shifts in the vector distribution from the primarily indoor-biting *Anopheles gambiae s.s.* and *Anopheles funestus* to the more zoophagic outdoor-biting and outdoor-resting *Anopheles arabiensis* [3] which is increasingly contributing to malaria transmission despite high LLIN and IRS coverage [4]. There is a need for interventions that target mosquito populations outside houses or at other points in the mosquito life cycle, as well as targeting insecticide-resistant populations [4–6].

All *Anopheles* mosquitoes (males and females) must periodically feed on liquid and sugars to survive — common sources include plant tissue and floral nectar. Mosquitoes are guided to sugar sources by chemical attractants. Attractive targeted sugar baits (ATSBs) attract mosquitoes to a source of liquid and sugar to deliver an ingestion toxicant that kills the mosquito [7]. These attract-and-kill strategies have been proven to reduce vector abundance in a limited number of trials [8], even in sugar-rich environments [9].

An ATSB, manufactured by Westham (Hod-Hasharon, Israel), was tested in Israel and Mali, where bait stations with a food dye marker (without toxicant) established that at least 35% of the mosquito population fed on the stations [10]. Proof-of-concept studies in Mali also demonstrated the ability of ATSB to reduce malaria vector populations when the ingestion toxicant dinotefuran was included in bait stations [10]. Studies in Mali have also established that two bait stations installed on opposite exterior walls of eligible structures at the height of 1.8 m were associated with at least 30% of target mosquitoes feeding on bait stations [11]. Studies conducted in Mali in 2017 showed ATSBs reduced mosquito density, the proportion of older females, the proportion of sporozoite-infected females, and the entomological inoculation rate (EIR). Together, these data strongly suggest that ATSBs can significantly reduce parasite transmission [10]. Modeling of ATSB suggests the potential to reduce mosquito populations across a range of settings even when used in addition to other indoor vector control tools [6, 12]. Durability studies conducted in Mali, Kenya, and Zambia in 2019 and 2020 showed that the Westham ATSB remains effective for at least 6 months (unpublished data, personal communication with J Entwistle).

ATSBs may be particularly valuable in mitigating insecticide resistance [13] because the ingestion toxicants are a different class of insecticides than the contact insecticides deployed near human sleeping spaces in LLINs or as part of IRS.

These trials aim to assess the impact of the Westham ASTB® on malaria morbidity when provided in addition to universal coverage with IRS and/or LLINs. Based on results from modeling studies it is hypothesized, based on previous studies in Mali [11], that deployment of ATSBs will result in a 30% or greater decrease in the malaria case incidence and parasite prevalence compared to universal vector control coverage alone (standard of care). Additional outcomes include the durability of ATSBs, entomological outcomes, community perceptions of ATSB, and human safety.

## Methods/design

The SPIRIT reporting guidelines were used in this protocol, with the checklist available as a supplemental file to the main manuscript [14].

### Study sites

**Fig. 1:**
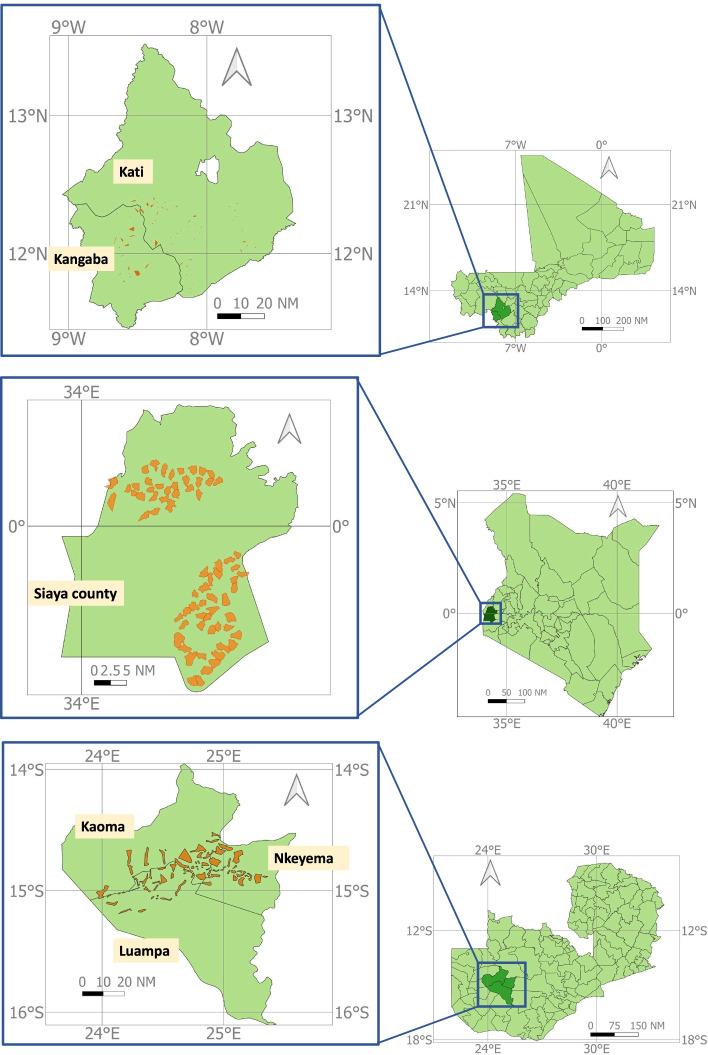
Maps of each study site in Mali, Kenya, and Zambia

Three similar stand-alone community (cluster) randomized controlled trials have been planned in southern Mali, western Kenya, and western Zambia. In Mali, the study site is in the health districts of Kangaba, Kati, and Ouelessebougou in the Kolikoro Region, approximately 60 km southeast of Bamako. In Kenya, the study site is in Siaya County, approximately 50 km west from Kisumu. In Zambia, the study site is in Western Province in the districts of Kaoma, Luampa, and Nkeyema (Fig. 1). Table 1 provides details of noted differences in the site-specific protocols between Mali, Kenya, and Zambia.

**Table 1:** Noted differences between the Mali, Kenya, and Zambia protocols

	Mali	Kenya	Zambia
Site location in each country	Health districts of Kangaba, Kati, and Ouelessebougou in the Kolikoro Region	Rarieda and Alego-Usonga sub-counties, of Siaya County	Kaoma and Nkeyema Districts, Western Province
Transmission and seasonality	Intense and highly seasonal (peak May–Nov)	Moderate–high year-around (peaks Jun–Jul and Nov–Dec)	Moderate–high (peak Jan–Jun)
Primary (and secondary) vectors [13, 15, 16]	*An. gambiae s.s.*, *An. coluzzii* (*An. arabiensis*)	*An. funestus*, *An. arabiensis* (*An. gambiae* s.s.)	*An. arabiensis*, *An. funestus* (*An. coustani*, *An. squamosus*, *An. rufipes*, *An. pretoriensis*)
ATSB deployment and replacement	ATSBs deployed continuously throughout 2-year trial (replaced at least every 6 months)	ATSBs deployed continuously throughout 2-year trial (replaced at least every 6 months)	ATSBs deployed seasonally during each year of the 2-year trial (Nov–Jun), replaced as needed
Standard of care	AL first-line treatment, universal coverage with LLINs, and SMC in children <5 years old	AL first-line treatment, universal coverage with LLINs, and RTS,S being piloted in 2/3 of the study area	AL first-line treatment, universal coverage with either LLINs or IRS
Total clusters (per arm)	76 (38)	70 (35)	70 (35)
Cluster formation	Single villages or groups of adjacent villages (100–400 households per cluster)	Single villages or groups of adjacent villages (100–400 households per cluster)	Clusters do not necessarily abide by village boundaries (250–350 households per cluster)
Buffer between area of cluster used in analyses	Not required	>1.2 km	>1.2 km
Age used for primary outcome of confirmed malaria incidence from cohort	5–<15 years	1–<15 years	1–<15 years
Cohort follow-up time periods	8-month seasonal cohort per year, totaling 16 months of follow-up during 2-year trial	12-month cohort per year, totaling 24 months of follow-up during 2-year trial (cohorts replaced every 6 months)	6-month seasonal cohort per year, totaling 12 months of follow-up during 2-year trial
Confirmation of two consecutive RDT positive tests during cohort follow-up visits to rule out HRP2 persistence	Microscopy	Microscopy	PCR
Statistical power for analysis of primary outcome of confirmed malaria incidence from cohort	90%	80%	80%
Timing of household surveys	Cross-sectional (peak transmission Oct–Nov)	Continuous (period)	Cross-sectional (peak transmission Mar–Apr)
Prevalence survey measure of infection	Microscopy, with PCR on negatives only	RDT, with dried blood spots available for PCR if desired	RDT, with dried blood spots available for PCR if desired

Transmission in Mali is intense and highly seasonal, with peak transmission from May to November (Table 1). The prevalence of *Plasmodium falciparum* infections by rapid diagnostic test (RDT) was 19% in children aged <5 years and reaches 30% in some areas [17]. Transmission in western Kenya is moderate to high year-round, with seasonal peaks in June to July and November to December. A continuous survey at the Kenya site found that the annual parasite prevalence by RDT in 2019 among children aged <5 years was 37%, and within the general population, it was 29%. Malaria transmission in western Zambia is moderate to high and seasonal, with peak transmission occurring from January to June. RDT parasite prevalence estimated from the baseline household survey conducted in April–May 2021 was 66.7% and 55.4% among children <5 years and all ages, respectively. Confirmed malaria incidence in the study area in Zambia during the peak transmission season January–June in 2019–2021 ranged from 415 to 641 confirmed cases per 1000 per year, according to the routine health information system.

The three sites vary in the species composition for malaria vectors (Table 1). The primary vectors are *An. gambiae s.s.* and *An. coluzzii* in Mali and *An. funestus* and *An. arabiensis* in western Kenya and western Zambia [13, 15, 16]. All study sites have documented pyrethroid resistance in their primary malaria vectors [18–20].

### Intervention: ATSBs

**Fig. 2:**
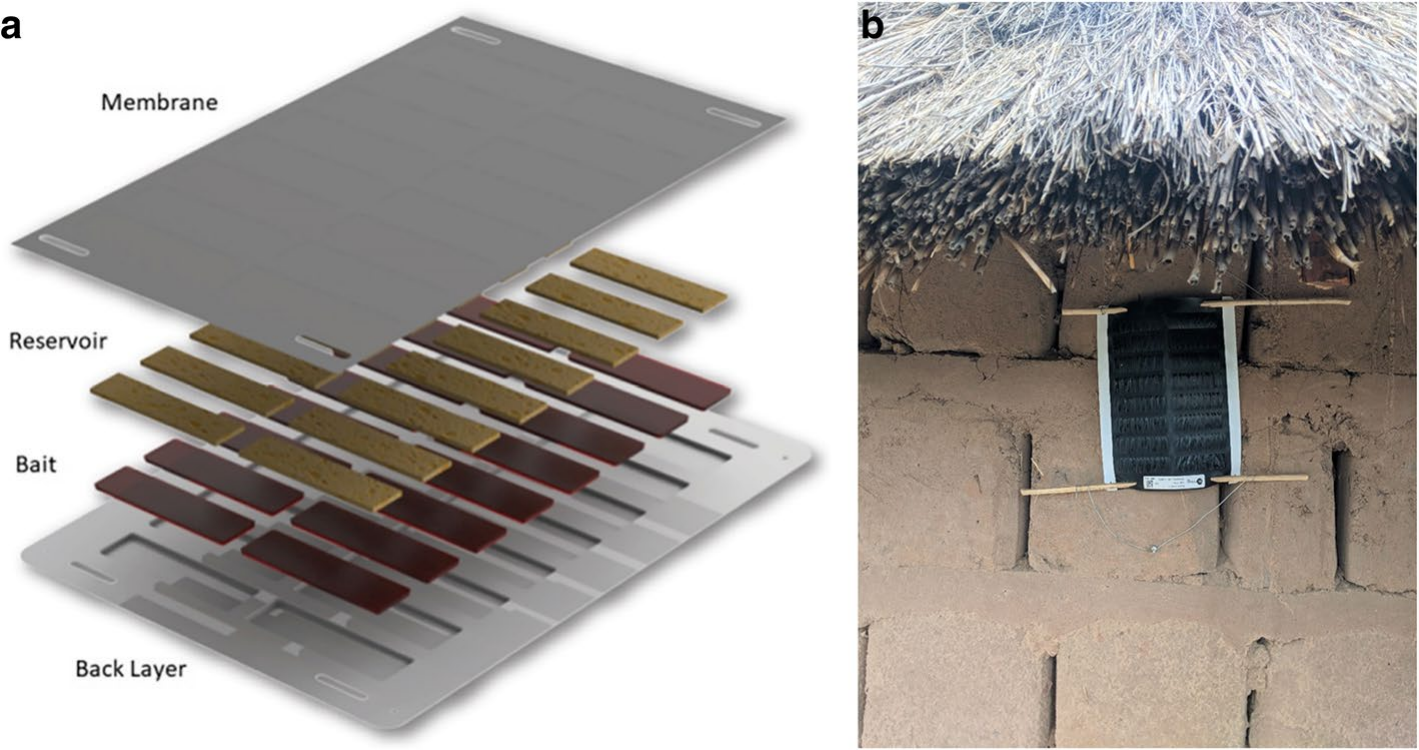
Photographs of the attractive targeted bait stations used in Mali, Kenya, and Zambia: **A** components of the ATSB, **B** a photograph of an installed ATSB in Zambia (November 2021)

The ATSB under study manufactured by Westham Co. (Hod-Hasharon, Israel) presents an attractant sugar meal consisting of fruit syrup laced with dinotefuran as the active ingredient to kill foraging vectors. Dinotefuran (N-methyl-N′-nitro-N″-[(tetrahydro-3-furanyl)methyl]guanidine) (MITSUI CHEMICALS, Inc.) is a neonicotinoid insecticide effective at rapidly killing mosquitoes (Fig. 2). ATSBs also contain a bittering agent, Bitrex® (Johnson Matthey), to deter human consumption. The bait station is designed to hold and protect the attractive bait from the environment by using a perforated membrane over a set of wells that hold the bait. Mosquitoes can probe and feed through pores in the membrane to access the sugar meal and the active ingredient. Environmental assessment and subsequent trials in Mali have demonstrated that when deployed within the ATSB, the toxicant poses limited risk to non-target organisms, including pollinators [11] and humans (ERM, 2021, unpublished ATSB Human Risk Assessment).

Durability studies have recently been completed. These studies involved installing the bait station on the exterior walls of structures and assessing bait station integrity over a 6–8-month period.

The ATSB is designed to provide population-wide protection by killing mosquitoes and thereby reducing their population size and longevity. Initial results from Mali show that high ATSB coverage is associated with a 70% reduction in the density of target vectors, a 97.1% reduction in female *Anopheles* with ≥3 gonotrophic cycles, and an overall younger age structure of the mosquito population, resulting in a 97.8% reduction in sporozoite rate and EIR [10, 21].

#### Deployment of ATSBs in intervention clusters and inclusion criteria

Two ATSBs will be installed per eligible structure. Structures are eligible to receive an ATSB if they meet the following criteria: a complete roof, walls at least 1 m high and wide, at least three complete exterior walls, the stations cannot be easily accessed by peri-domestic animals or children, the structure is not under active construction, and the structure is not a working platform (e.g., used for food drying or pot drying). Stations will be mounted on the outside of exterior walls, with the perforated membrane facing outwards, at least 1 m off the ground, ideally immediately under the eaves where possible.

A cadre of trained monitoring assistants will provide information to households on the ATSB. ATSBs will be installed subject to informed consent by the householder. Bait stations will be marked with a unique barcode to allow tracking over time and association with specific household information. In Mali and Kenya, ATSBs will be deployed continuously for the 2-year trial, replaced every 6 months, or earlier if they are damaged or removed (Table 1). In Zambia, ATSBs will be deployed seasonally each year of the trial from November to June. In all three countries, ATSBs will be replaced if damaged or removed. Used or damaged ATSBs will be disposed of in high-temperature incinerators appropriate for insecticide-contaminated waste.

Prior to and throughout ATSB deployment, community sensitization will be conducted through community meetings, posters, pamphlets, or other printed materials. Qualitative research has been conducted in the study sites, including stakeholder mapping, in-depth interviews, and focus group discussions, whose results will feed into the community sensitization and mobilization strategies.

### Standard of care/control

The standard of care for malaria prevention in Mali includes universal coverage with LLINs as well as seasonal chemoprevention for malaria for children under five (SMC). The standard of care in Zambia is universal vector control coverage with either LLIN coverage or IRS. The standard of care in Kenya includes universal LLIN coverage. In approximately two-thirds of the study area in Kenya, children aged 6 to 12 months of age are eligible to receive the RTS,S/AS01_E_ malaria vaccine which is being routinely implemented by the Kenya National Vaccines and Immunizations Programme through a WHO-sponsored pilot as a four-dose schedule at 6, 7, 9, and 24 months of age (https://www.who.int/initiatives/malaria-vaccine-implementation-programme). All sites use a combination of RDTs and microscopy for diagnosing malaria in the health system, and all sites use artemether-lumefantrine (AL) as the first-line treatment for uncomplicated malaria. All sites provide community case management for malaria for children aged < 5 years.

### Study design

Each of the three stand-alone trials will be an open-label, two-arm, cluster-randomized, controlled trial (cRCT) design comparing ATSB + universal vector control against universal vector control alone. A cluster trial design is indicated given the intended community-level effect of ATSBs on malaria transmission. Each trial is independently powered.

The trial design incorporates adaptive elements. Planned adaptations for this trial include sample size refinement based on parameters estimated from baseline cohorts and cross-sectional surveys, including baseline incidence, prevalence, coefficient of variation (CV), loss to follow-up, and non-response. Secondly, the adaptive design will include an interim analysis of the primary endpoint of clinical malaria incidence [22], with a single interim analysis after the first year of the trial in Mali and Zambia and two interim analyses at approximately 50% and 75% person-time accrual in Kenya.

#### Cluster formation and contamination mitigation

In Mali and Kenya, single villages, or groups of adjacent villages, form clusters consisting of on average 149 households in Mali and 100–400 households in Kenya. In Zambia, communities of approximately 250–350 households in close proximity to one another will form clusters; these clusters do not necessarily abide by administrative boundaries (Table 1).

ATSB will be deployed to all households in the intervention clusters, but in Kenya and Zambia, sampling of participants for outcome ascertainment will be limited to a core area within each study cluster to minimize the risk of contamination bias due to any spill-over effect. Based on the community effect observed in insecticide-treated mosquito net trials [23], the minimum distance between the households included in the primary analyses within intervention and control clusters will be 1.2 km apart. The exact boundaries for the core sampling areas and buffer areas will be determined during cluster formation and structure enumeration. In Mali, where villages are more defined and spread out, there will be no division of clusters into core and buffer areas. Instead, a cluster will be formed with a minimum separation of 1150 m of uninhabited space between clusters to minimize potential contamination.

#### Randomization

Clusters will be allocated in a 1:1 ratio using restricted randomization to minimize imbalances between the intervention and control arms of key predictors of the primary outcome. These may include clinical malaria incidence, malaria prevention intervention coverage, population size, housing density, RTS,S vaccine administration areas, and others. Random selection of a specific allocation sequence (randomization) and assignment of arms to intervention or control will be conducted separately for each site by an independent statistician.

### Primary outcome measures

The primary outcome is the incidence of clinical malaria, defined as a history of fever within the previous 48 h or a measured temperature of ≥37.5°C, plus a positive malaria RDT in cohorts aged 1–14 years in Kenya and Zambia and 5–14 years in Mali. These outcomes will be ascertained during monthly follow-up visits or through cohort participant sick visits.

Key secondary outcomes include:

1. Prevalence of *P. falciparum* infection among participants aged 6 months and older in Zambia and Mali (among participants aged 1 month and older in Kenya), detected by RDT in cross-sectional surveys
2. Incidence rate of passively reported confirmed malaria among participants of all ages, defined as the number of confirmed malaria cases (by RDT or microscopy) detected at health facilities (Mali and Kenya only)
3. Entomological outcomes, including daily female vector survival, vector species abundance, composition, sex, sporozoite rate, EIR, and resistance to dinotefuran, among other insecticides
4. Social and behavioral outcomes related to the acceptability and correct knowledge of ATSBs
5. Cost of ATSB deployment and incremental cost-effectiveness ratios
6. Human safety and adverse events

### Data collection procedures for measurement of outcomes

For all data collection procedures involving human subjects, written informed consent and assent (where age-appropriate) will be obtained. An overview of the planned data collection activities can be found in Fig. 2.

#### Baseline data collection

Baseline estimates of clinical malaria incidence, malaria prevalence, within-cluster homogeneity, loss to follow-up, and refusal will be ascertained. The baseline measures will be used to revise sample sizes prior to the trial, develop and refine data collection instruments, organize data collection logistics, assess where additional quality control measures are required, and provide data for restricted randomization.

#### Longitudinal cohorts

Mali will have two 8-month seasonal cohorts, totaling 16 months of follow-up time (Table 1). In Kenya, which has perennial transmission, four 6-month cohorts will be conducted to cover the full 2-year period. Zambia will have two 6-month seasonal cohorts, totaling 12 months of follow-up time.

Mapping and census exercises were conducted in each trial site to generate a household- (Kenya and Zambia) or individual-level (Mali) sampling frame. In Kenya, a mapping and census exercise will be repeated within 2 months before each cohort is enrolled to update the sampling frame. Cohort households will be selected via simple random sampling (SRS) from the household enumeration sampling frame within the core area of clusters in Kenya and Zambia. One individual meeting the inclusion criteria will be selected via SRS to participate in the cohort from each selected household at any given time. In Mali, eligible children will be directly sampled from census lists for each cluster.

At the enrollment visit in Mali and Zambia, information about the households will be captured, including LLIN ownership and use, housing characteristics, and household demographic information. In Kenya, enrolment will occur at study clinics, and separate visits will be made to the households to collect data on housing characteristics. All cohort participants will be cleared of malaria parasite infection at enrollment with artemether-lumefantrine. In Zambia and Mali, confirmation of parasite clearance by blood smear will be ascertained 2 weeks after enrollment. In Kenya, RDTs will be conducted at the enrollment visits. Confirmation of parasite clearance will be ascertained by blood smear or RDT 2 weeks after enrollment (if RDT at enrolment was positive, a blood smear will be performed and if RDT at enrolment was negative, an RDT will be performed). Those found positive at the clearance confirmation visit will be treated and removed from the cohort.

Cohort participants will then be seen monthly. In Mali and Zambia, these visits will be conducted at the household. In Kenya, these will be conducted at study clinics. At each scheduled visit, a dried blood spot (DBS) will be collected on filter paper. Study participants with a history of fever in the last 48 h or current fever (axillary temperature of ≥ 37.5° C) will have an RDT performed (in Mali, a blood smear will be taken for those that are RDT positive; in Kenya, if the participant reports recent malaria infection within 5 weeks, because of persistence of HRP-2 antigenemia following malaria infections, a blood smear will be taken instead of an RDT). Those without fever or history of fever will not have an RDT performed. A child with fever plus a positive RDT test (or positive blood smear) will be considered to have clinical malaria and contribute to the numerator of the primary outcome measure. Episodes of uncomplicated clinical malaria will receive the standard of care ACT treatment. Children with severe malaria will be referred for treatment with parenteral artesunate. Participants who are treated for malaria will have 2 weeks of follow-up time censored from the subsequent follow-up period due to the prophylactic effect of the standard of care anti-malarial.

Among all participants with a positive RDT in Mali, a repeat RDT will be conducted during the next monthly scheduled visits to confirm parasite clearance (no clearance confirmation will be conducted in Kenya and Zambia). If the RDT remains positive, the individual will be treated again. Due to the persistence of HRP-2 antigenemia following malaria infections, however, determination of whether there is an active malaria infection for the purpose of the primary outcome will be ascertained by microscopy in Mali and by PCR in Zambia.

Inclusion criteria include currently living in the study household and aged 1–14 years (Zambia and Kenya) or 5–14 years (Mali) at the time of enrollment. Exclusion criteria comprise the following: household location/location of residence located in a buffer area or outside all cluster boundaries, pregnant at the time of enrollment, detection of patent malaria parasitemia 2 weeks after initial parasite clearance dose, ill and needing referral to the nearest health center at time of enrollment (Mali and Zambia), those for which AL is contraindicated, known sickle cell disease and taking daily cotrimoxazole prophylaxis (Kenya), and current enrollment in another interventional study (Kenya). Pregnant women are excluded because they are eligible for monthly intermittent preventative therapy (IPT). Any participant found to become pregnant at any time during the cohort study will be omitted from further follow-up and referred to the nearest clinic for antenatal care.

#### Cross-sectional household surveys

Cross-sectional household surveys will be used to estimate parasite infection prevalence among participants aged 6 months and older in Zambia and Mali and among participants aged 1 month and older in Kenya. In Zambia and Mali, three cross-sectional household surveys will be conducted to estimate point parasite prevalence during peak malaria transmission season (October–November in Mali and April in Zambia), at baseline and in years 1 and 2 of trial implementation (Table 1). In Kenya, where transmission is year-round, a continuous “rolling” household survey will capture the 12-month period parasite prevalence.

In Mali, individuals meeting the inclusion criteria will be selected via SRS from census enumeration lists for each cluster. In Zambia and Kenya, households will first be selected from the core sampling area of each cluster. In Kenya, every member of the household will be included, whereas in Zambia, one individual meeting the inclusion criteria will be selected via SRS to participate in the blood draw from each selected household. Consenting/assenting individuals will be tested for circulating malaria antigens using a finger prick blood sample for an RDT. Individuals with a positive RDT will be treated with an ACT according to national policy. The household surveys will also capture information about malaria intervention coverage, health care seeking behavior, housing characteristics, household demographic information, and ATSB knowledge and perceptions. These data will be collected by administering a standardized questionnaire to the household head or other appropriate respondents for each selected household.

#### Passive case detection in Mali and Kenya

Confirmed passive malaria case incidence will be calculated from data from public health facilities and community health workers operating under each facility. The population denominator for incidence calculations will be derived from the census enumeration listing conducted prior to the trial start in each cluster. In Mali, facility-based assistants will be provided with electronic tablets with a custom application for collecting case data. Training will be provided for dispensary personnel and community health workers (CHWs) to capture case data. Data will be transmitted weekly to the field data manager. In Kenya, health care data are entered into routine Ministry of Health registers that have been converted into ScanForm (https://about.scanform.qed.ai/) registers at facilities and by CHWs, which capture individual-level patient data that are then captured through a photo via a Smartphone App and are converted to a working database. Data will be transmitted at least once per month.

#### Rapid ethnographic data

The qualitative component of this study is designed to understand potential factors that influence vector control coverage, including ATSB and LLIN. Focus group discussions (FGD) with community members and ATSB monitoring assistants and in-depth interviews (IDI) with community members will be conducted in intervention areas to understand the potential factors influencing ATSB and LLIN acceptability and coverage. Early results will be used to guide community engagement before the first ATSB deployment and inform strategies to ensure high community engagement and coverage levels throughout the trial.

#### Cost data

ATSB product, delivery, and deployment costs will be collected to estimate the financial and economic costs of the intervention. Cost data collection will include a review of program records and reports, invoices, budgets, expenditure reports, and interviews with intervention implementers. Interviews with trial staff will be focused on resource use during the implementation of the study interventions.

#### Entomological data collection

Entomological monitoring activities will take place in the three trial sites using indoor and outdoor CDC UV light traps and human landing catches. The mosquito collections will be conducted in 16 clusters in Kenya, 24 clusters in Mali, and 20 clusters in Zambia. The clusters will be equally assigned to each study arm. Entomological monitoring in Zambia will be aligned with the deployment of ATSBs and will be carried out seasonally, while in Mali and Kenya, entomological monitoring will be carried out year-round. Specimens will be used to estimate key indicators including daily female vector survival (parity as determined by ovarian dissection), vector abundance, sporozoite rate, and calculated EIR. Specimens will also be used to monitor species composition and sex as well as indoor and outdoor landing rates. Insecticide resistance for dinotefuran and insecticides used in LLINs and IRS will be monitored annually in a subset of clusters. Samples of bait stations will be routinely collected from the field (and replaced) for laboratory testing to confirm ongoing target vector attraction and susceptibility to the bait stations.

#### Human safety monitoring

Adverse events (AEs) and severe adverse events (SAEs) will be monitored through both passive and active data collection systems. AEs will be collected for those related to AL for treatment of uncomplicated malaria in the cohort, as well as exposure to the ATSBs in the general community (non-ingestion). An SAE will be considered any death of a cohort participant, or any ingestion of the bait from an ATSB in the general community.

### Sample size and statistical power

The trials will include 76 clusters (38 per arm) in Mali and 70 (35 per arm) in each of Kenya and Zambia. All sample size estimates for epidemiological measures are based on the formula for community randomized control trial designs as outlined in Hayes and Moulton [24]. All parameters, as described below and in Tables 2 and 3, use existing data on the incidence rate, CV, and estimated rates of loss to follow-up. The sample sizes are subject to change following the collection of baseline data on the nuisance parameters used in the sample size calculations (incidence rate, ICC and CV values, and loss to follow-up).

**Table 2:** Assumptions, parameters, and sample size estimations for the longitudinal cohorts in Mali, Kenya, and Zambia

	Mali	Kenya	Zambia
Clusters per arm (overall)	38 (76)	35 (70)	35 (70)
Trial duration in calendar years (seasonality of FU) (total FU per participant time in months)	2 years (8-month seasons) (16 months FU)	2 years (12-month seasons) (24 months FU)	2 years (6-month seasons) (12 months FU)
*α* (type 1 error probability for 2-year trial)	0.05 (Haybittle-Peto with one interim analysis)	0.05 (Haybittle-Peto with two interim analyses at approx. 50% and 75%)	0.05 (Haybittle-Peto with one interim analysis)
Power	88%	80%	80%
Baseline incidence of clinical malaria in the target age group	0.40 events per person year (based on 0.6 incident events during an 8-month malaria season) (5y–<15y)	0.845 events per person year during a 12-m malaria transmission season (1y–<15y)^a^	0.50 events per 6-month malaria season (Jan–Jun) (1y–<15y)
Reduction in baseline incidence (incidence rate ratio = 0.70)	30%	30%	30%
Coefficient of variation	0.40	0.40	0.40
Assumed loss of person-time, including true LTFU plus loss due to exclusion of person-time following each treatment with AL	20%	20%	34%
Total person-years required (number enrolled per cluster before loss to follow-up)	**3850** person-years (obtained by enrolling 38 individuals per cluster to account for loss to follow-up, followed for total of 16 months)	**1260** person-years (obtained by enrolling 13.5 individuals per cluster to account for loss to follow-up, followed for total of 24 months)	**1610** person-years (obtained by enrolling 35 individuals per cluster to account for loss to follow-up, followed for total of 12 months)

^a^The observed event rate in this age group was 1128 per 1000 person-years in the control arm of a recently completed mass test-and-treat trial in this area. A more conservative event rate of 845/1000 will be used to account for an anticipated 25% reduction in clinical malaria in children 1–<5 years of age (28.6% of the sample study cohort) due to the implementation of the RTS,S/AS01_E_ vaccine in two-thirds of the study area (resulting in an estimated 7.4% reduction in event rates in children 1–<15 years), plus a further 17.6% reduction in malaria due to unforeseen changes in environmental factors, or boosting of other malaria control measures such as the scaling up of integrated community-based case management

**Table 3:** Assumptions, parameters, and sample size estimations for the cross-sectional surveys in Mali, Kenya, and Zambia

	Mali	Kenya	Zambia
Cluster per arm	38	35	35
*α* (2-tailed)	0.05	0.05	0.05
Power	90%	80%	90%
Baseline parasite prevalence measured by RDT among participants age 6 months and older	50%	29.0%^b^	50.0%
Reduction in baseline prevalence	30%	30%	30%
ICC = intracluster correlation coefficient (coefficient of variation)	0.16 (cv = 0.4)	0.05	0.10
Non-response	20%	20%	20%
Sample size per cluster (before non-response)	25 (32)	24 (30)	16 (20)
Total sample size per survey round/year (before non-response)	1900 (2432)	1680 (2100)^a^	1120 (1400)

^a^Per year in Kenya as it uses a continuous survey approach. ^b^In Kenya the prevalence estimates are for individuals aged ≥1 month

#### Longitudinal cohort

A sample size of 3850 person-years is required in Mali, 1260 person-years in Kenya, and 1610 person-years in Zambia, to detect a 30% reduction in the primary outcome allowing for an overall 5% type-1 error rate following Haybittle-Peto boundaries allowing for interim analyses, and after accounting for the coefficient of variation (CV) of 0.4 (Table 2). An anticipated loss to follow-up of person-time of 20% will be used in Mali and Kenya which accounts for cohort members dropping out from data collection. A higher rate of 34% loss to follow-up will be used in Zambia based on baseline data. In Mali, the trial will have 88% power for the case incidence outcome, while 80% will be used in Kenya and Zambia.

#### Cross-sectional household surveys

The sample size calculations for the cross-sectional surveys are based on detecting at least a 30% reduction in the *Plasmodium falciparum* prevalence in each site at each survey/year with 90% power in Mali and Zambia and 80% power in Kenya (Table 3). Based on preliminary data for the baseline prevalence and CV (or ICC) of the cluster-specific parasite prevalence, the estimates of sample size after accounting for site-specific anticipated non-response are as follows: a total of 32 individuals per cluster per survey in Mali (25 individuals per cluster after non-response), 30 per cluster per year in Kenya (24 individuals per cluster after non-response), and 20 per survey in Zambia (16 individuals per cluster after non-response).

### Statistical analysis

The primary analysis of treatment effect for the primary outcome of clinical incidence of malaria and the secondary outcome of parasite prevalence will be based on intention-to-treat at the individual level without adjustment for anticipated confounding variables. For all analyses, statistical models will include a random intercept for study cluster to account for correlated observations at the cluster level due to the community randomized design.

A single interim analysis of the primary endpoint is planned in Mali and Zambia after the first transmission season regardless of the total number of events. In Kenya, the interim analyses will be either event or time driven, depending on which comes first, occurring after approximately 50% and 75% of person-time has accrued or after 50% (*n* = 372) or 75% (*n* = 558) number of expected primary outcome events have occurred in the control arm. The number of events will be tracked by an independent statistician not involved in the trial. Following the Haybittle-Peto rule, the interim analysis will be considered significant if *p* < 0.001 and the final null-hypothesis significance testing will be conducted with alpha levels of 0.05 in Mali and Zambia (single interim analysis) and at 0.049 in Kenya (two interim analyses) [25, 26]. The DSMB will make final decisions for the trial in the event that the interim analysis shows a significant result in favor of the ATSBs.

The primary outcome analysis of the incidence rate of clinical malaria in the cohort will use a multi-level random intercept (variance components) model constructed on a generalized linear model framework with a Poisson likelihood and a log link function. The study arm will be included as a fixed effect independent variable.

*P. falciparum* infection prevalence among participants aged 6 months (Mali and Zambia) or 1 month (Kenya) and older detected by RDT in the household surveys will be analyzed using a multi-level random intercept (variance components) model constructed on a generalized linear model framework with a Bernoulli likelihood and a logit link function. The study arm will be included as a fixed effect independent variable.

### Quality assurance during the trial

Data quality monitoring will be conducted to ensure that the trial is continuously operated according to protocol. The analysis will consist of a blinded review of all datasets generated for quality and completeness. Continuous corrective actions will occur based on these data, as needed.

### Trial governance

The ATSB project is governed by a steering committee with members from the Bill & Melinda Gates Foundation, IVCC (Innovative Vector Control Consortium), Westham Co., PATH, the Liverpool School of Tropical Medicine, the University of Bamako, and representatives from IVCC’s external expert advisors. An independent Data Safety and Monitoring Board (DSMB) has been established for each study site. The primary responsibilities of the DSMB are to periodically review and evaluate the accumulated study data for participant safety, study conduct and progress, and, when appropriate, efficacy. The DSMBs are responsible for making recommendations to investigators and research ethics committees concerning the trials. The DSMBs are empowered to advise to stop the trial early if there is evidence of harm or mismanagement.

### Study timeline

**Fig. 3:**
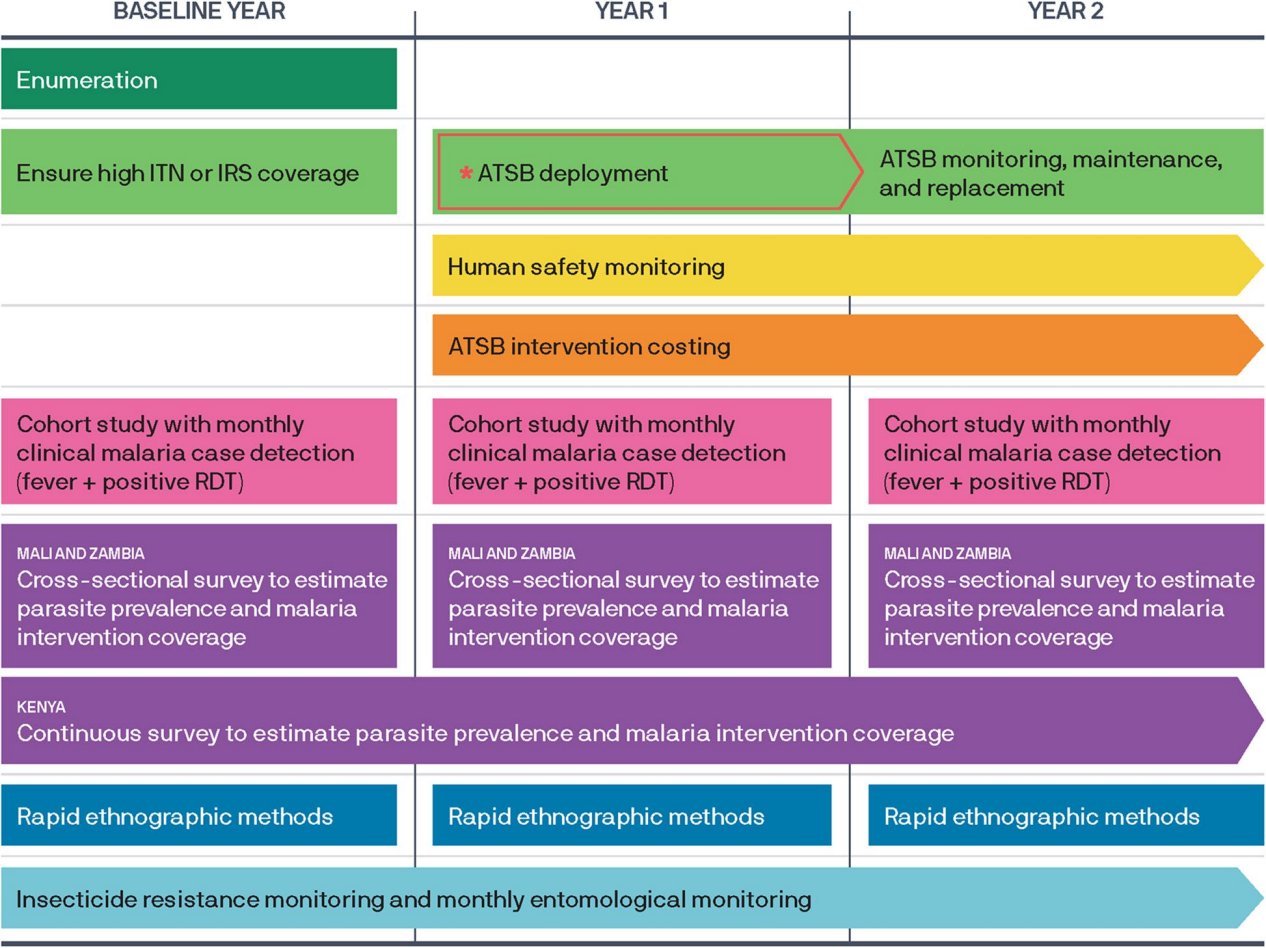
Overview of the data collection plan in intervention and control areas

The projects will take place over 3 years, with the trials occurring over 2 years and the baseline conducted during the first year (see Fig. 3 for a data collection overview and Tables 4, 5, and 6 for a detailed participant timeline). Restricted randomization will take place following baseline data collection before ATSB deployment. ATSBs will then be deployed at least 2–6 weeks before the start of the longitudinal cohorts and every 6 months thereafter in Kenya and Mali. Continuous entomological data collection will be conducted from baseline and through years 1 and 2 post-ATSB deployment in Mali. Kenya will have continuous entomological data collection through year 1. Entomological data collection will be conducted only during the peak transmission season Jan–Jun of each year in Zambia.

**Table 4:** Detailed study timeline for Mali

Study activity	2021			2022				2023				2024	
	Q2	Q3	Q4	Q1	Q2	Q3	Q4	Q1	Q2	Q3	Q4	Q1	Q2
Baseline cohort enrollment^a^	**X**												
Baseline cohort^a^		**X**	**X**										
Baseline household survey^a^			**X**										
Ethnographic data collection^a^			**X**	**X**			**X**	**X**			**X**	**X**	
Randomization of clusters to ATSB or control				**X**									
ATSB deployment, monitoring, and replacement					**X**	**X**	**X**		**X**	**X**	**X**		
Adverse event monitoring					**X**	**X**	**X**	**X**	**X**	**X**	**X**	**X**	
Cohort enrollments^a^					**X**				**X**				
Cohort monthly follow-ups^a^						**X**	**X**			**X**	**X**		
Household surveys^a^							**X**				**X**		
Interim analysis of cohort data								**X**					
Cost data collection								**X**	**X**	**X**	**X**		
Entomological monitoring	**X**				**X**	**X**	**X**		**X**	**X**	**X**		
Data analyses												**X**	**X**
Data dissemination												**X**	**X**

^a^Informed consent and assent obtained

**Table 5:** Detailed study timeline for Kenya

Study activity	2021			2022				2023				2024		
	Q3	Q4	Q1	Q2	Q3	Q4	Q1	Q2	Q3	Q4	Q1	Q2	Q3	Q4
Baseline cohort enrollment^a^	**X**													
Baseline cohort^a^		**X**	**X**											
Ethnographic data collection^a^		**X**	**X**	**X**			**X**							
Randomization of clusters to ATSB or control			**X**											
ATSB deployment or replacement			**X**		**X**		**X**		**X**					
ATSB monitoring				**X**	**X**	**X**	**X**	**X**	**X**	**X**	**X**			
Adverse event monitoring			**X**	**X**	**X**	**X**	**X**	**X**	**X**	**X**	**X**			
Cohort enrollments^a^			**X**		**X**		**X**		**X**					
Cohort monthly follow-ups^a^				**X**	**X**	**X**	**X**	**X**	**X**	**X**	**X**			
Household surveys^a^				**X**	**X**	**X**	**X**	**X**	**X**	**X**	**X**			
Passive surveillance			**X**	**X**	**X**	**X**	**X**	**X**	**X**	**X**	**X**			
Cost data collection			**X**	**X**	**X**	**X**	**X**	**X**	**X**	**X**	**X**			
Entomological monitoring		**X**	**X**	**X**	**X**	**X**	**X**							
Data analyses												**X**	**X**	**X**
Data dissemination													**X**	**X**

^a^Informed consent and assent obtained

**Table 6:** Detailed study timeline for Zambia

Study activity	2020		2021				2022				2023		
	Q4	Q1	Q2	Q3	Q4	Q1	Q2	Q3	Q4	Q1	Q2	Q3	Q4
Baseline cohort enrollment^a^	**X**												
Baseline cohort^a^		**X**	**X**										
Baseline household survey^a^			**X**										
Ethnographic data collection^a^			**X**	**X**			**X**	**X**			**X**	**X**	
Randomization of clusters to ATSB or control				**X**									
ATSB deployment, monitoring, and replacement					**X**	**X**	**X**		**X**	**X**	**X**		
Adverse event monitoring					**X**	**X**	**X**	**X**	**X**	**X**	**X**	**X**	
Cohort enrollments^a^					**X**				**X**				
Cohort monthly follow-ups^a^						**X**	**X**			**X**	**X**		
Household surveys^a^							**X**				**X**		
Interim analysis of cohort data								**X**					
Cost data collection								**X**	**X**	**X**	**X**		
Entomological monitoring	**X**				**X**	**X**	**X**		**X**	**X**	**X**		
Data analyses												**X**	**X**
Data dissemination												**X**	**X**

^a^Informed consent and assent obtained

### Ethical approvals and protection of human subjects

Ethical approval in Zambia has been obtained from the National Health Research Ethics Board (NHREB) at the University Teaching Hospital, the PATH Research Ethics Committee, and the Institutional Review Board at Tulane University. For the Mali trial, ethics review was undertaken by the Comite D’Ethique of the University of Sciences, Techiques and Technologies of Bamako and by the Ethics Committee of the London School of Hygiene and Tropical Medicine. For the trial in Kenya, ethical approval has been obtained from the Kenya Medical Research Institute (KEMRI) Scientific and Ethics Review Unit (SERU), the Liverpool School of Tropical Medicine; the Centers for Disease Control and Prevention IRB is operating on a reliance agreement with the KEMRI SERU. Protocol amendments will be sought for each study site for all relevant ethics committees if a protocol change is made.

Individuals greater than 18 years of age will provide individual consent. For individuals aged 6 months to less than 18 years of age, consent will be sought from the parent or guardian of the child. For children greater than 6 years and less than 18, oral assent will be sought from the child. All concent and assent will be ascertained by trained data collectors.

Confidentiality will be ensured through removal of all uniquely identifiable data. All data stored and managed on password-protected electronic platforms

## Discussion

These three trials are designed to establish the efficacy of ATSBs for the prevention of clinical malaria in a range of transmission settings in malaria-endemic Africa. Results will be used to inform national and global health policy regarding the use of the ATSB for malaria vector control.

The use of three stand-alone but standardized cRCTs in different geographic areas with differing vectorial systems, seasonal patterns, human-built environments, vegetation patterns, and cultures will ensure that the results of the three trials will have high levels of external validity for sub-Saharan African settings. The use of restricted randomization, ITT analyses, measurement of endpoints along the causal pathway for the intervention, and other rigorous quality control methods will ensure the study’s internal validity. Analyst and investigator blinding will also strengthen internal validity. Finally, the use of buffer zones or adequate separation between clusters will also enhance the study’s internal validity by reducing contamination that could bias results towards the null effect.

There are several potential threats to the internal validity of the trial. First, the use of sham ATSBs deployed in control clusters was deemed infeasible, and thus, the trial will not be blinded to the study participants. Potential bias due to the absence of blinding will be partially offset through highly standardized protocols for entomological field procedures. Laboratory processing of mosquito and human blood specimens will be blinded. Second, the potential for changes in human behavior in intervention clusters (or control clusters) affecting seeking treatment for fevers and malaria control intervention coverage, namely LLIN use, will be minimized through comprehensive community engagement and communications in all clusters. Additionally, study teams will monitor LLIN use in intervention and control areas to document changes throughout the course of the trial. There is always the potential for error in the measurement of outcomes, exposure to the interventions, and potential confounding factors in large and complex trials. We have gone to considerable lengths to minimize such errors with standardized outcomes and data collection instruments, electronic data capture systems for all fieldwork, and robust diagnostic methods for ascertaining disease and infectious status in humans.

The trial results will be submitted to the World Health Organization (WHO) Vector Control Advisory Group (VCAG) for review of the public health value of the ATSB. Should VCAG determine that the ATSB class has public health value, a vector control guidelines development group (GDG) would subsequently develop a recommendation around the use of products in this class. These recommendations may include guidelines for a pathway for second-in-class products to achieve WHO prequalification status, which may require entomological data demonstrating non-inferiority to the first-in-class product [27]. A WHO policy recommendation and product prequalification are essential steps towards scale-up and to facilitate countries and donors to purchase and deploy ATSBs.

## Trial status

In Mali, the most recent version of the protocol was approved on 20 April 2021 by the ethical institution of record the Comite D’Etique of the Universite des Sciences, des Techniques et des Technologies de Bamako (Ref # 2021/124/CE/USTTB). Baseline recruitment for the seasonal cohort took place in the month of May 2021; enrolled participants were followed up for 8 months (through January 2022). Recruitment for the first seasonal cohort post-ATSB deployment took place in the month of May 2022; enrolled participants are currently being followed up for 8 months (through January 2023). The second season cohort is planned to take place from May 2023 through January 2024, with enrollment of participant conducted in the first month of the cohort. In Kenya, the latest protocol approval is for an amendment on 25 February 2022 by the ethical institution of record the Scientific Ethics Review Unit at the Kenya Medical Research Institute (Ref # KEMRI/CGHR/368/4189). Recruitment for the baseline cohort took place during the month of August 2021 with those recruited followed up for 5 months. Recruitment for the main trial cohort study took place in the months of April–May 2022; enrolled participants will be followed up for 24 months with new participants enrolled each 6 months (i.e., enrollment occurring over a 1-month period every 6 months). In Zambia, the latest protocol approval is for an amendment on 27 November 2021 (Ref # 1197-2020) by the ethical institution of record the National Health Research Ethics Board (NHREB) at the University Teaching Hospital. Recruitment for the baseline season cohort took place in the month of December 2020; enrolled particiapnts were followed up through June 2021. Recruitment for the first follow-up seasonal cohort took place in the month of December 2021; enrolled participants were followed up for 6 months through June 2022. The second season cohort is planned to take place in January–June 2023, with recruitment taking place in December 2022.

## Supplementary Information

**Additional file 1.** SPIRIT checklist.

## Data Availability

The datasets analyzed during the current study will be made available from the corresponding author and/or the study site principal investigator upon reasonable request.

## Abbreviations

ACT: Artemisinin-based combination therapy
AL: Artemether-lumefantrine
ANC: Antenatal care
ATSB: Attractive targeted sugar bait
CDC: (US) Centers for Disease Control and Prevention
CHW: Community health worker
cMIS: Continuous Malaria Indicator Survey
CRCT: Cluster-randomized control trial
CS ELISA: Circumsporozoite protein enzyme-linked immunosorbent assay
DBS: Dried blood spot
DHIS2: District health information system II
DSMB: Data safety monitoring board
EIR: Entomological inoculation rate
GPS: Global positioning system
HH: Household
FGD: Focus group discussion
ICC: Intracluster correlation coefficient
ICER: Incremental cost-effectiveness ratio
IDI: In-depth interview
IPTp: Intermittent preventative treatment in pregnancy
IRS: Indoor residual spray
ITT: Intention-to-treat
IVCC: Innovative Vector Control Consortium
KAP: Knowledge, attitudes, and practices
LLIN: Long-lasting insecticide-treated net
MACEPA: Malaria Control and Elimination Partnership in Africa
MPAG: Malaria Policy and Advocacy Group
NIH: National Institute of Health
PCR: Polymerase chain reaction
RDT: Rapid diagnostic test
SRS: Simple random sampling
UV: Ultraviolet
VCAG: Vector Control Advisory Group
WHO: World Health Organization
